# Supplementary material for: An adaptive synaptic array using Fowler–Nordheim dynamic analog memory
Source: Nat Commun. 2022 Mar 29;13:1670. doi: 10.1038/s41467-022-29320-6 (PMC8964701; doi:10.1038/s41467-022-29320-6)
Supplement: Supplementary file 1 — Supplementary Information [file 41467_2022_29320_MOESM1_ESM.pdf]

Supplementary Information

I. Circuit implementation and programming of FN-DAM

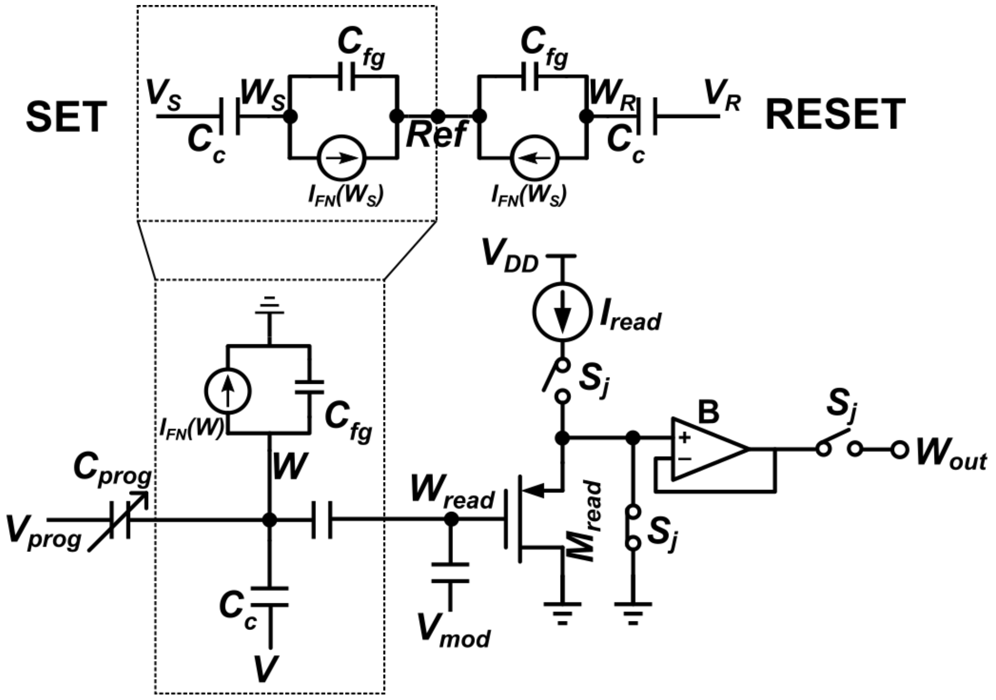

Figure 1: Circuit implementation of the FN-DAM cell with read-out and programming circuitry.

Each FN-DAM cell comprises of two dynamical systems labeled as SET and RESET as shown in Fig. 1(e) and reproduced in SI Figure 1. Each of these dynamical systems comprises of two floating-nodes: a tunneling-node (W) that stores the dynamical state; and a readout-node (W_{read}) that is used to read the dynamical state through a decoupling capacitor C_{read} and a read-out PMOS transistor M_{read}, as shown in SI Figure 1. Each of these cells can selected for READ and PROGRAMMING using the switch S_j. For this work, the selection of S_j is performed using a serial shift-register but a row-column decoder configuration could also have been used for faster access. When a cell is selected for READ using the terminal W_{out}, the read-out transistor M_{read} is configured as a source-follower using a current-source I_{read} (located in the periphery of the FN-DAM array) and the voltage of the source follower is buffered using B. The initial charge on each of the floating-nodes (SET or RESET) can be programmed individually using a combination of tunneling (to increase charge, coarse tuning) and hot-electron injection (to decrease charge, fine tuning). Details of such programming has been described in [1, 2] and here we describe some of the programming parameters. The tunneling gate W, which stores the dynamic analog memory, is first biased in the FN-tunneling regime. By setting V_{prog} to a high-potential of 22 V (using an internal or external charge-pump), the tunneling node is pushed to ~8 V which is sufficient to initiate observable FN-tunneling across the gate-oxide (approximately 13 nm for work). Note that W_{read} is capacitively decoupled from the tunneling node to avert readout disturbances. The readout node is also biased at a lower voltage to prevent injection into the readout node during operation. The potential of the readout node is lowered through hot-electron injection. Hot-electron injection is initiated by setting V_{DD}= 7 V and the input pin to a value such that the

drain-to-source voltage across M_{read} is above 4.2 V. The switch S_j allows for individual control of each FN-DAM block for reading and programming.

II. Read-disturbance characterization

To reduce the effect of read disturbance, in our implementation we have capacitively decoupled the readout circuit from the memory as shown in the Supplementary Information Figure 1. We conducted read-disturbance experiments, where twelve FN-DAM memory elements were randomly accessed every minute for 1000 cycles and the relative change in weight was measured after every read. The measured result shown in Supplementary Information Figure. 2 verifies that read-disturb in our implementation of FN-DAM is random and the magnitude is less than the precision of the update and measurement.

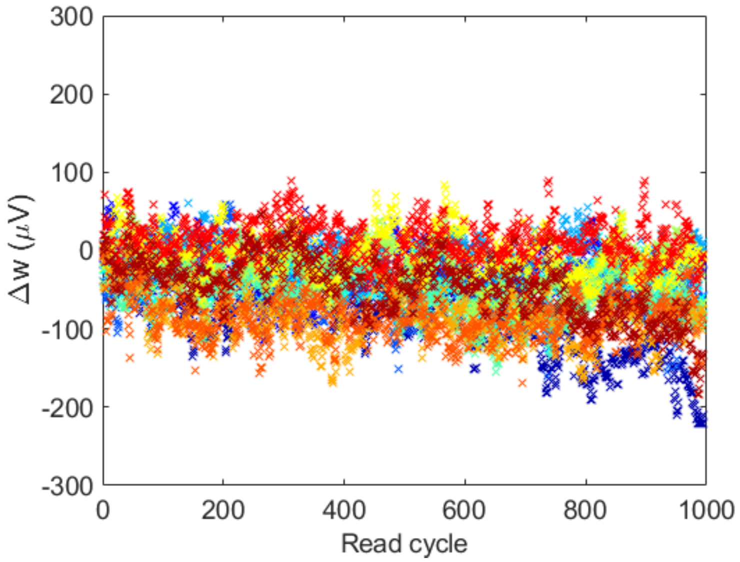

Figure 2: Read disturbance reflected as change in weight parameters measured from 12 FN-DAM devices over 1000 read cycles. Each color in the figure represents one FN-DAM device.

III. Write Energy Dissipation Estimation

The magnitude of input pulse required, $V_{train}(t)$ (SI Fig. 3a) so that the floating gate node at current potential $V_{FG}(t)$ shifts to a target voltage V_T is given by:

$$V_{train}(t) = \frac{V_T - V_{FG}(t)}{C_R}$$

Where C_R is the input capacitive coupling ratio $C_R = \frac{C_C}{C_C + C_{FG}}$. The floating gate voltage $V_{FG}(t)$ is approximated by the following dynamic [1]:

$$V_{FG}(t) = \frac{k_2}{\log(k_1 t + k_0)} \quad (1)$$

The energy required to charge the input capacitor is given as

$$E(t) = \frac{1}{2} C_{in} \left(V_{in}(t)\right)^2$$

SI Fig. 3b shows instantaneous energy required to charge unit capacitance when $V_T = 7.6V$ and $V_{FG}(0) = 7.5V$. The input capacitance of our device was 1 pF, and the instantaneous write energy per update increased from 5 fJ to 2.5pJ over 12 days.

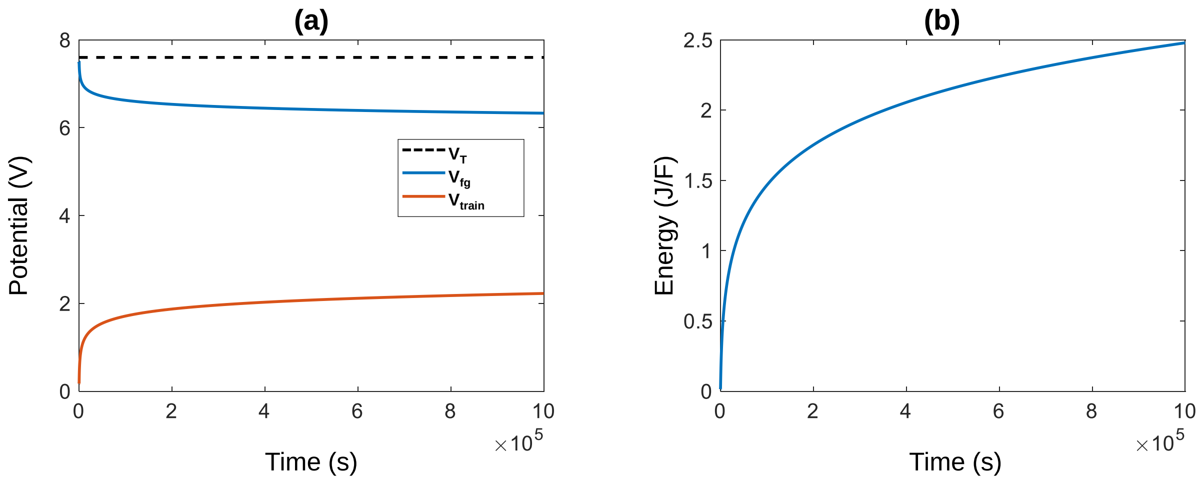

Figure 3: a) Target voltage, floating gate voltage and training voltage as a function of time. B) Energy required to charge unit capacitance as a function of time.

IV. Memory Retention

SI Fig. 4a shows retention times for different T (25°C, 60°C, 100°C) estimated using the retention model equations (19)-(21). These models have been verified using experiments conducted at 100^oC. SI Fig. 4b shows the measured results where the weights stored in 12 FN-DAM devices kept at 100^oC were measured over a duration of 15 hours. Note that compared to standard reliability testing of non-volatile memories where the chipsets are baked offline and then the retention measurements could be performed under standard operating temperature, for testing FN-DAM, the data needs to be measured continuously under high temperature condition. This is because FN-DAM is a dynamical memory which stores information in the degree of temporal desynchronization between two dynamical systems. Therefore, performing continuous measurements under 250°C operating condition would have require use of temperature compensated read-out circuits. However, the required reliability information can also be inferred from continuous measurements at 100°C, shown in SI Fig. 4b. The baseline drift due to the memory read-out circuits were first calibrated during the first 400 min and used to zero out the dynamical response of each of the FN-DAM device. Then, at 400 min time instant a SET pulse (3.3V for 1 second duration) was applied

to all the memory devices which programmed all the device to a specific memory state. The degree of desynchronization was continuously measured and is plotted in SI Fig. 4b. The resynchronization process is accurately predicted by the model at $100^{o}C$ (SI Fig. 4b inset)

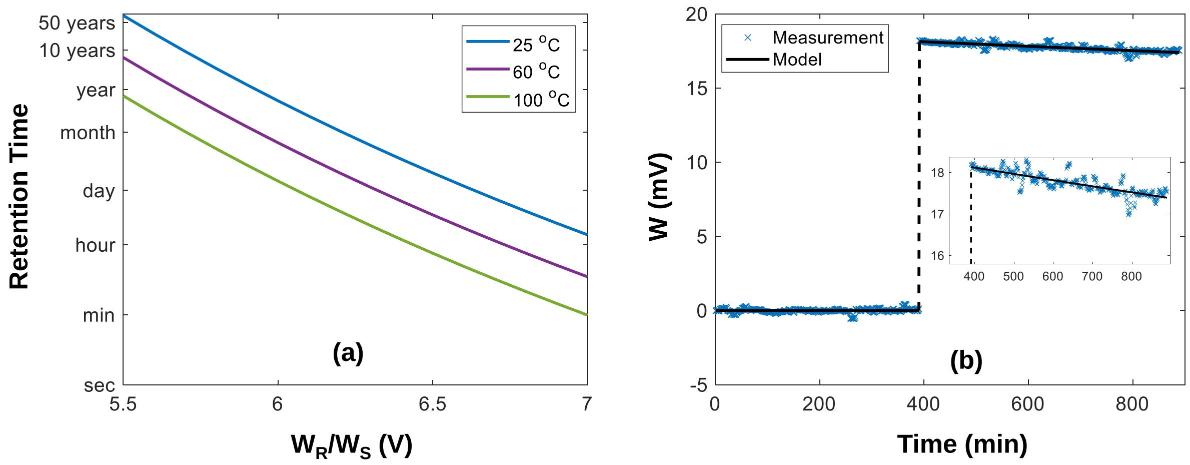

Figure 4: a) Simulated retention time as a function of SET/RESET node voltage for different operating temperatures. b) Measurement and modeling results from 12 FN-DAM devices desynchronized at 100 °C.

V. Read Energy Dissipation

The readout power is dependent on the readout accuracy required and the speed at which it operates.

For a PMOS in a source follower configuration, the readout noise is given by:

$$V_n^2 = \frac{4kT}{g_m}\Delta f = \frac{4kT}{q} * \frac{q}{g_m}\Delta f = \frac{4U_T q}{g_m}\Delta f$$

For subthreshold operation,

$$g_m = \frac{\kappa I_d}{U_T}$$

$$\therefore V_n^2 = \frac{4U_T^2 q}{\kappa I_d}\Delta f = \frac{4U_T^2 q V_{DD}}{\kappa P_{read}}\Delta f$$

Above equation is plotted in SI Figure 5 for different noise floors and readout frequency for $V_{dd} = 5V$, $U_T = 26\,mV$ and $\kappa = 0.7$

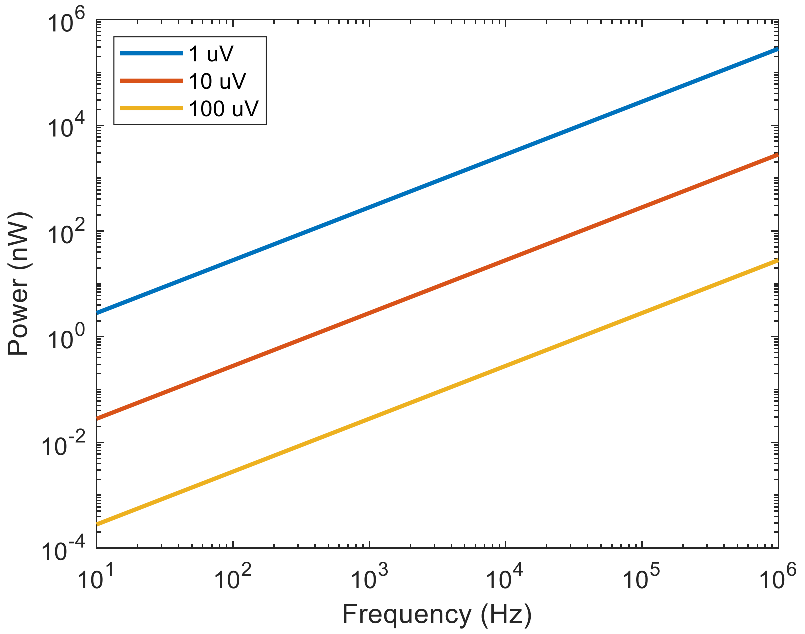

Figure 5: Minimum power required to read floating gate voltage as a function of required readout speed. Noise floors shown in legend.

VI. Programming dynamics

The FN-DAM is programmed by applying a pulse of magnitude $V_{train}(t)$ so that the node reaches a potential of V_T through the input coupling capacitor, as derived in the previous section. The programming ratio is given by:

$$\frac{\mathrm{I}_{\mathrm{prog}}}{\mathrm{I}_{\overline{\mathrm{prog}}}} = \frac{I_{FN}(V_T)}{I_{FN}\big(V_{FG}(t)\big)}$$

Dynamics of FN tunneling current are given by [1]:

$$\frac{I_{FN}\big(V(t)\big)}{C_T} = \frac{d\big(V(t)\big)}{dt} = \left(\frac{k_1}{k_2}\right) V^2 \exp\left(-\frac{k_2}{V}\right)$$

$$\frac{\mathrm{I}_{\mathrm{prog}}}{\mathrm{I}_{\overline{\mathrm{prog}}}} = \left(\frac{V_T}{V_{FG}(t)}\right)^2 \exp\left(\frac{k_2}{V_{FG}(t)} - \frac{k_2}{V_T}\right)$$

Above equation is plotted for 3 values of k_1 in SI Fig 6. which affect the dynamics of $V_{FG}(t)$. The parameter k_1 can be altered during the design phase by changing the area and capacitance of the floating gate node.

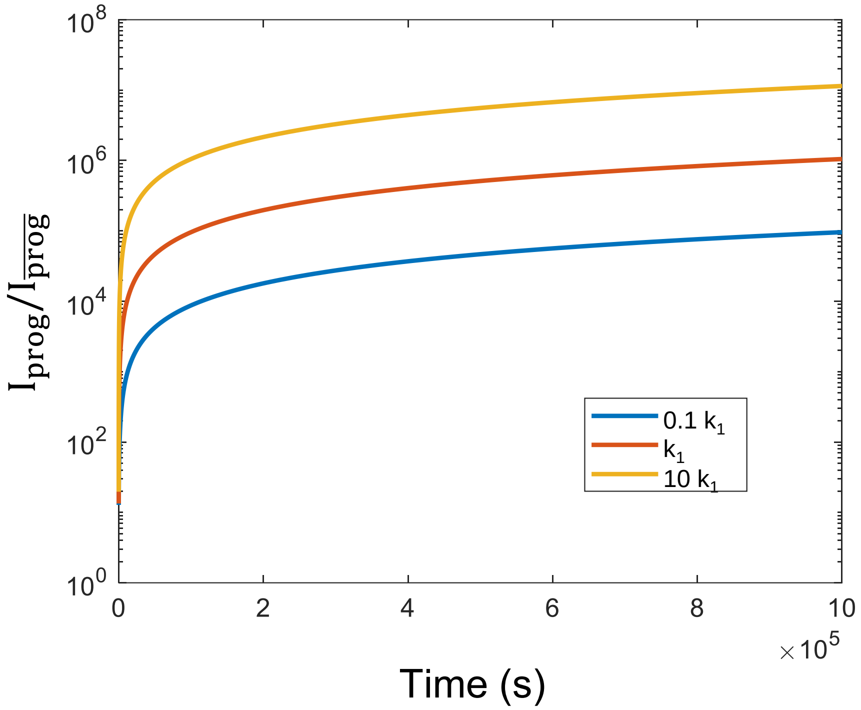

Figure 6: Programming ratio for different k_1 parameter which can be controlled by changing the size of tunneling junction.

VII. MLP and CNN architecture and training parameters

The neural network used for experiments with the Fisher-Iris dataset, is a three-layer multi-layer perceptron as shown in SI Fig. 7a. which also shows the magnitude of the weights obtained after training, as indicted by the thickness of the edges between the neurons across different layers. The blue edge represents an excitatory weight, and a red edge represents an inhibitory weight. SI Fig. 7b. shows the confusion matrix computed for one training run and for the entire Fisher-Iris dataset (150 data points).

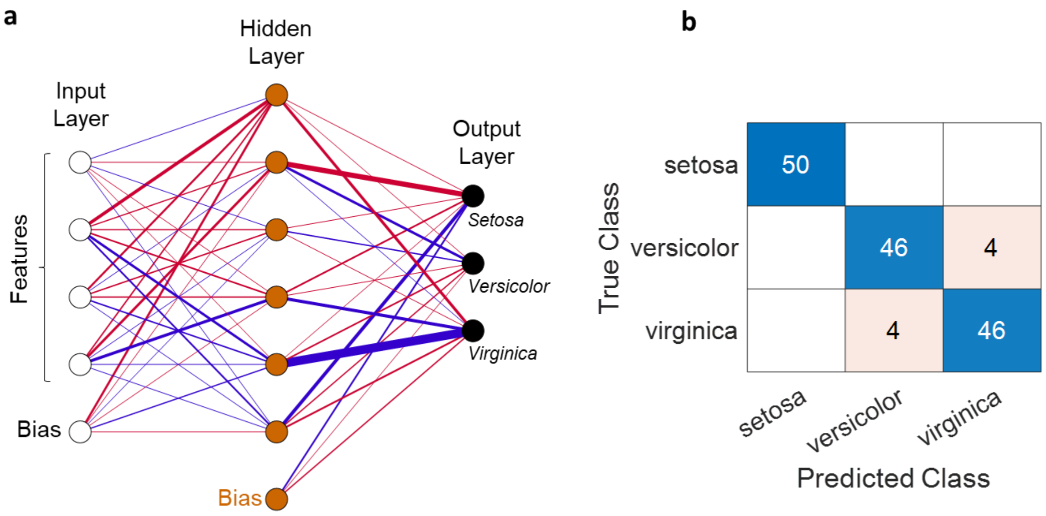

Figure 7: a) Experimentally trained network on Fisher Iris dataset. Thickness of connections between units indicate magnitudes of learned weights. Blue (red) connection indicates positive (negative) weight. b) Confusion matrix with for Fisher Iris dataset.

The convolutional neural network used for the MNIST experiment comprised of the following layers

**Layer**	**Name**	**Description**	**Activations**	**Parameters**
**1**	Image Input	28×28×1 images	28 x 28 x 1	0
**2**	Convolution	20 5×5×1 convolutions with stride 1	24 x 24 x 20	520
**3**	Batch Normalization	Batch normalization with 20 channels	24 x 24 x 20	40
**4**	ReLU	ReLU	24 x 24 x 20	0
**5**	Convolution	20 3×3×20 convolutions with stride 1	24 x 24 x 20	3620
**6**	Batch Normalization	Batch normalization with 20 channels	24 x 24 x 20	40
**7**	ReLU	ReLU	24 x 24 x 20	0
**8**	Max	Pooling	12 x 12 x 20	0
**9**	Convolution	40 3×3×20 convolutions with stride 1	12 x 12 x 40	7240
**10**	preluLayer	Parametric ReLU with 40 channels	12 x 12 x 40	40
**11**	Convolution	20 3×3×40 convolutions with stride 1	12 x 12 x 20	7220
**12**	Batch Normalization	Batch normalization with 20 channels	12 x 12 x 20	40
**13**	ReLU	ReLU	12 x 12 x 20	0
**14**	Fully Connected	10 fully connected layer	10	28810
**15**	Softmax	softmax	10	0

The network was constructed in MATLAB using Deep Learning toolbox and was trained using Stochastic Gradient Descent with Momentum. Only the weights in the Fully Connected layer were updated during training. SI Fig. 8 shows the confusion matrix with recognition accuracy obtained for each class of digits.

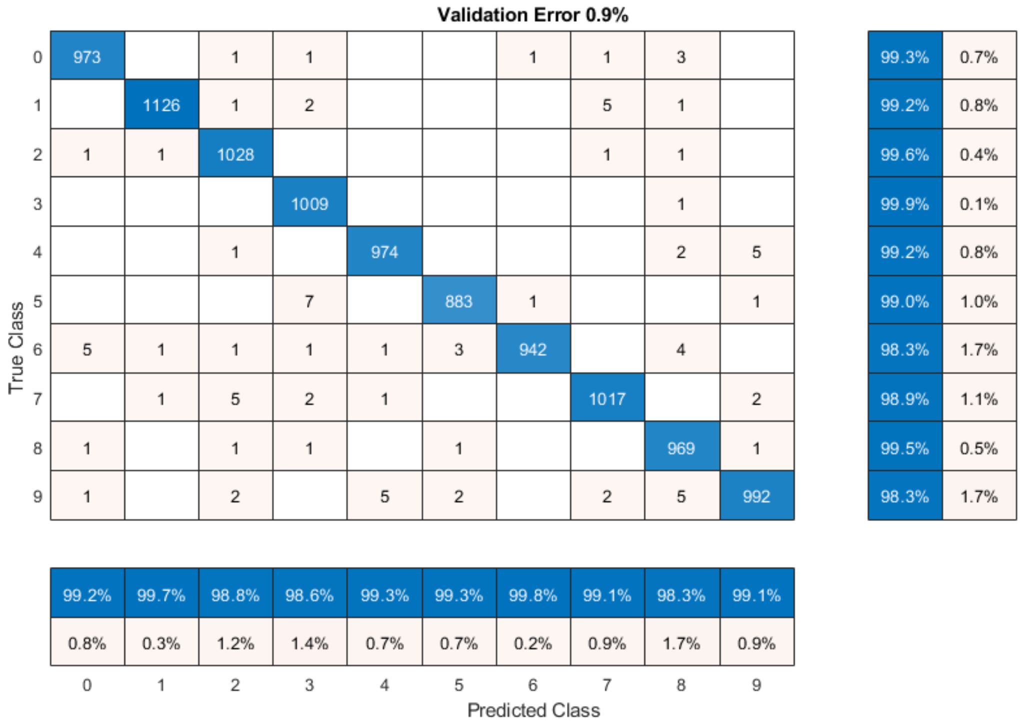

Figure 8. Confusion matrix with for simulated CNN implementation of FN-DAM on MNIST dataset.

VIII. Retention of MLP parameters

The data retention times for FN-DAM cells (or the cells' volatility) is a smooth function with respect to the dynamic state of the memory (SET and RESET voltages). This is also shown in SI Fig. 4a. During training the FN-DAMs are biased to operate in the volatile region where lower data retention is traded-off for lower energy consumption. Once the neural network has been trained, it is not necessary for the system to reach a non-volatile regime. Because of the differential architecture of the FN-DAM, both the SET and RESET nodes discharge/decay down to the slow-tunneling regime and at a discharge-rate that is approximately constant across all the memory cells. In this case, the performance of the learning algorithm (neural network) that normalizes the weights should remain robust with minimal degradation in recognition accuracy. To verify this, we trained a neural network, shown in SI Fig. 7(a) on the Fisher's Iris dataset using FN-DAM as storage for network parameters. Post-training, we transferred our chip into a baking oven which was set to 225ºC. Note that this is the maximum temperature setting for the baking oven (Quincy lab Model 40) set at 225 ºC. Care was taken in transporting the chipsets to prevent any electrostatic discharge (ESD) issues. After 6 hours of baking, the chips were taken out, weights were read-out and the classification accuracy of the network was measured. SI Fig. 9 a below compares the weights stored on the FN-DAM cells, before and after baking. The result in SI Fig. 9 b. shows that even though all the post-bake weights exhibit a decay with respect their pre-bake values, but when normalized ($\mathbf{w}_{norm} = \mathbf{w}/||\mathbf{w}||_1$), both the pre-bake and post-bake values remain relatively invariant.

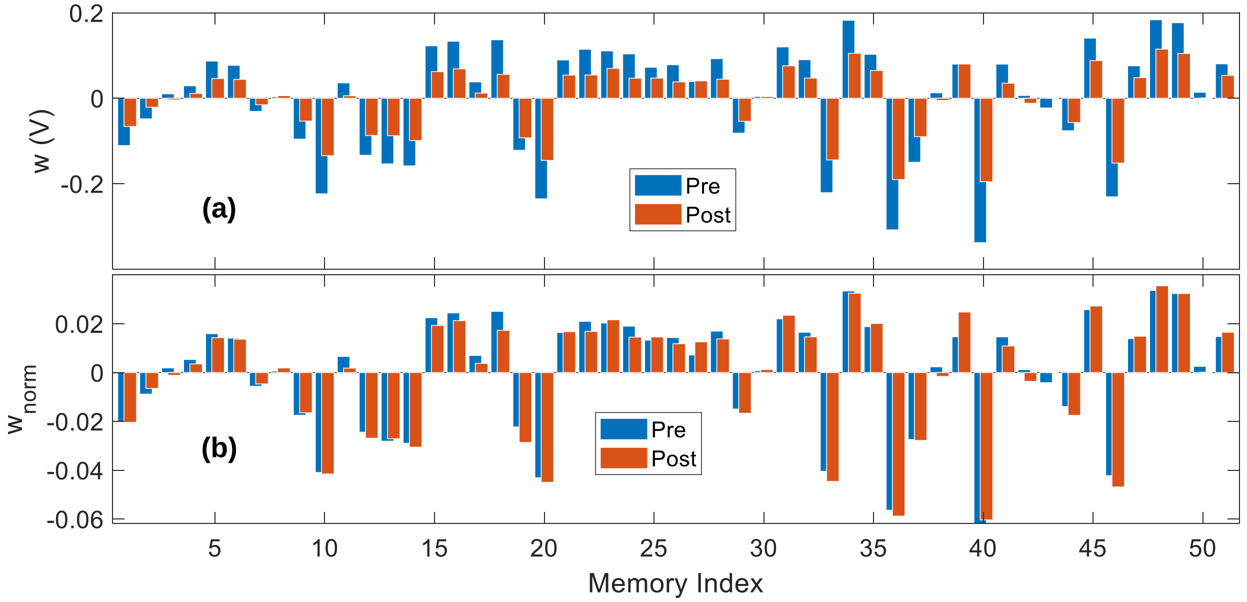

Figure 9. (a) *Weights stored on FN-DAM memory cells after training a neural network on the Fisher-Iris dataset, before baking (Pre) and after baking (Post); and (b) normalized weights before baking and after baking.*

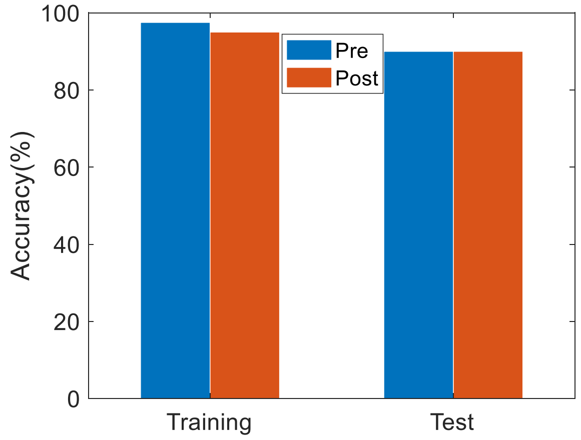

Figure 10. Training and test accuracy obtained using the pre-bake and post-bake values of weights stored on the FN-DAM.

SI Fig. 10. compares the training and test accuracy obtained using weights stored on the FN-DAM before baking and after baking. The result shows that while the training accuracy reduces nominally (97.5% to 95%), the test accuracy remains unchanged. Note that after the bake, the respective SET and RESET voltages (W_S and W_R) decays such that the FN-DAM enters the high-retention regime.

References

1. L. Zhou and S. Chakrabartty, "Self-powered timekeeping and synchronization using fowler–nordheim tunneling-based floating-gate integrators," *IEEE Transactions on Electron Devices*, vol. 64, no. 3, pp. 1254–1260, 2017.

2. D. Mehta, K. Aono, and S. Chakrabartty, “A self-powered analog sensor-data-logging device based on fowler-nordheim dynamical systems,” *Nature communications*, vol. 11, no. 1, pp. 1–9, 2020.
